# CXCR7 Is Involved in Human Oligodendroglial Precursor Cell Maturation

**DOI:** 10.1371/journal.pone.0146503

**Published:** 2016-01-07

**Authors:** David Kremer, Qiao-Ling Cui, Peter Göttle, Tanja Kuhlmann, Hans-Peter Hartung, Jack Antel, Patrick Küry

**Affiliations:** 1 Department of Neurology, Medical Faculty, Heinrich-Heine-University, Düsseldorf, Germany; 2 Montreal Neurological Institute, McGill University, Montreal, Quebec, Canada; 3 Institute of Neuropathology, University Hospital Münster, Münster, Germany; Instituto Cajal-CSIC, SPAIN

## Abstract

Differentiation of oligodendroglial precursor cells (OPCs), a crucial prerequisite for central nervous system (CNS) remyelination in diseases such as Multiple Sclerosis (MS), is modulated by a multitude of extrinsic and intrinsic factors. In a previous study we revealed that the chemokine CXCL12 stimulates rodent OPC differentiation via activation of its receptor CXCR7. We could now demonstrate that CXCR7 is also expressed on NogoA- and Nkx2.2-positive oligodendroglial cells in human MS brains and that stimulation of cultured primary fetal human OPCs with CXCL12 promotes their differentiation as measured by surface marker expression and morphologic complexity. Pharmacological inhibition of CXCR7 effectively blocks these CXCL12-dependent effects. Our findings therefore suggest that a specific activation of CXCR7 could provide a means to promote oligodendroglial differentiation facilitating endogenous remyelination activities.

## Introduction

Multiple Sclerosis (MS) is a chronic inflammatory demyelinating disease of the human central nervous system (CNS), leading to gradual degeneration and loss of myelin sheaths and oligodendrocytes. As a consequence, axonal function is impaired and axons are severely damaged [1]. Although repair activities are limited within the adult CNS, remyelination can be observed as a result of resident oligodendroglial precursor cell (OPC) activation particularly in early disease stages. These cells can be recruited into MS lesions where they differentiate into functional myelinating cells [2]. However, due to a blockade of oligodendroglial differentiation remyelination efficiency remains overall poor [3–6]. Neutralization of inhibitory cues or activation of stimulatory pathways could therefore be a viable strategy to enhance CNS remyelination. Chemokines are highly conserved among mammalian species and regulate a plethora of different physiological processes, such as, for instance the modulation of cell-cell interactions, immune cell chemotaxis and developmental processes in a variety of tissues including the brain [7, 8]. Chemokines bind mainly to G-protein coupled receptors and exert strong effects in neuroinflammatory diseases [9, 10]. In the past the impact of chemokines on the survival and behavior of OPCs has been under closer investigation [11, 12]. Particularly CXCL12 (stromal derived growth factor 1; SDF-1) has been described as a relevant factor for the behavior of oligodendroglial precursors [13] and the regulation of blood-brain-barrier integrity in neuroinflammation [14]. In a previous study we have identified the CXCL12 receptor CXCR7 as a potent mediator of OPC differentiation in the inflamed rodent brain [15]. We could now translate our results to the human paradigm confirming that signaling through CXCR7 leads to increased human oligodendroglial precursor cell differentiation suggesting that specific activation of this receptor could be a novel therapeutic approach to promote endogenous remyelination activities.

## Material and Methods

### Isolation, culture and immunocytochemistry of fetal hOPCs

Human fetal CNS tissue obtained from 15 to 18 gestational week embryos was provided by the Human Fetal Tissue Repository (Albert Einstein College of Medicine, Bronx, NY). This developmental stage precedes CNS myelination. Human fetal OPCs (hOPCs) as A2B5 expressing cells were isolated immuno-magnetically as previously described [16]. The purified cells were plated on poly-L-lysine-coated plastic coverslips (Nunc, Rochester, NY). The cultures were grown in Dulbecco’s modified essential medium (DMEM)-F12 supplemented with N1 (Sigma, Oakville, ON), 0.01% bovine serum albumin (BSA), 1% penicillin-streptomycin, B27 supplement (Invitrogen, Burlington, ON), thyroid hormone (T3, 2 ng/ml, Sigma, Oakville, ON), platelet-derived growth factor AA (PDGF-AA, 10 ng/ml, Sigma, Oakville, ON) and basic fibroblast growth factor (FGF2, 10 ng/ml, Sigma, Oakville, ON). All tissue samples were obtained under protocols approved by the McGill University institutional review boards. Fetal material was obtained from the Albert Einstein School of Medicine fetal repository program with written consents obtained at that site. All experiments were conducted in accordance with the Helsinki Declaration. Additional studies were performed using human fetal OPCs and respective media purchased from 3H Biomedical (Uppsala, Sweden) and cultured according to the manufacturer’s protocol.

To assess the effects of CXCL12 stimulation on hOPCs (myelin marker expression, morphological maturation) fetal hOPCs were cultured for up to 12 days in defined media supplemented with recombinant human CXCL12 (100ng/ml in PBS buffer supplemented with 0.1% bovine serum albumin; R&D Systems, Minneapolis, MN) and growth factors (10ng/ml BDNF, Calbiochem, San Diego, CA and 10ng/ml IGF, MJS BioLynx, Brackville, ON) or growth factors alone. Medium was changed on days 3, 6 and 9. CCX771 (ChemoCentryx, Mountain View, CA) was reconstituted in DMSO according to the manufacturer’s instructions and used at a concentration of 10 nM in a 30 min pre-treatment step on hOPC cultures prior to the addition of CXCL12 at the above-described concentration. Immunocytofluorescent staining was performed using the following primary antibodies: hybridoma anti-O4 IgM antibody (1:50; Montreal Neurological Institute, McGill University, Montreal, Quebec, Canada and [17]), hybridoma anti-GalC IgG_3_ antibody (1:50; Montreal Neurological Institute, McGill University, Montreal, Quebec, Canada and [18]) and mouse anti-2‘,3‘-cyclic nucleotide 3‘-phosphodiesterase antibody (CNPase; 1:1000; Sternberger Monoclonals, Lutherville, MD). For visualization IgM-FITC or TxR (1:100; Jackson ImmunoResearch, Westgrove, PA), IgG3-FITC or TxR (1:100; Biosource, Camarillo, CA) and IgG1 Alexa Fluor 488 (1/500; Molecular Probes, Leiden, the Netherlands) were used. Data are presented as mean +/- standard error of the mean (SEM) and significance was assessed by either two-sided Student’s t-test (unpaired comparison for means) or one-way ANOVA (GraphPad Prism). Experimental groups were considered significantly different at *p<0.05, **p<0.01, ***p<0.001; ns, not significant.

### Immunohistochemistry

We retrospectively investigated 12 brain biopsy tissue samples from 12 MS patients (9 women, 3 men; age 21 to 74 years, mean age 44 +/- 16 years) and six control cases (3 women, 3 men; age 25 to 72, mean age 56 +/- 8 years; see Table 1). All lesions fulfilled the generally accepted criteria for the diagnosis of MS. The tissue samples showed the characteristics of active lesions with loss of myelin, infiltration by numerous phagocytes and fewer lymphocytes as well as gliosis. We classified the de- and remyelinating activity as described earlier [19]. Actively demyelinating lesion areas (AD; n = 7) were located at the plaque border, these areas were partially demyelinated and infiltrated by numerous macrophages containing myelin degradation products, such as myelin basic protein (MBP) or CNPase within their cytoplasm. Demyelinated lesion areas (DM) were infiltrated by macrophages and T cells, but macrophages did not contain myelin degradation products (biopsies; n = 2). In remyelinating areas (RM; biopsies; n = 3), thin, irregularly formed myelin sheaths were seen. Periplaque white matter (PPWM; biopsies; n = 7) showed no signs of demyelination. Additionally, tissue samples from six control patients without CNS pathology besides mild microglia activation and reactive gliosis were analyzed. Tissue specimens were fixed in 4% paraformaldehyde and embedded in paraffin. Tissue samples were cut in 4 μm thick sections and stained with hematoxylin/eosin (Merck, Darmstadt, Germany). Immunohistochemical staining was performed using a biotin-streptavidin peroxidase protocol (Dako, Glostrup, Denmark). After deparaffinization intrinsic peroxidase activity was blocked by incubation with 5% H_2_O_2_ in PBS for 20 min. Non-specific antibody binding was inhibited with 10% FCS in PBS for 25 min. Sections were pre-treated in a steamer with citrate (pH 6.0) prior to incubation with the primary antibody. 3,3'-diaminobenzidine (DAB) was used as a chromogen and sections were counterstained using hematoxylin. As primary antibodies we used rabbit anti-CMKOR1 (1:200; Proteintech, Chicago, USA), rabbit anti-CXCR7 (1:100; Millipore), rabbit anti-Nogo-A (1:750; Chemicon International, Temecula, CA), mouse anti-Nkx2.2 (1:100; Developmental Studies Hybridoma Bank, University of Iowa, Iowa) and mouse anti-Nogo-A (1:15.000; 11c7, a generous gift from M.E. Schwab, Brain Research Institute, University of Zürich and Department of Biology, Swiss Federal Institute of Technology Zürich, Switzerland). General tissue characterization (data not shown) was carried out using Luxol-fast blue (Sigma), mouse anti-KiM1P (1:5000; H.-J. Radzun, Department of Pathology, University of Göttingen, Germany), mouse anti-CD3 (1:25; Dako, Glostrup, Denmark), rabbit anti-Olig2 (1:300; IBL, Spring Lake Park, Minnesota), rabbit anti-GFAP (1:2000; Dako, Glostrup, Denmark), rabbit anti-MBP (1:1000; Boehringer Mannheim, Mannheim, Germany) and mouse anti-GFAP (1:50; Dako, Glostrup, Denmark). Double immunofluorescent staining was performed using rabbit anti-CXCR7 (see above), and mouse anti-NogoA (see above) or mouse anti-Nkx2.2 antibodies followed by Cy3 (1:200; Jackson ImmunoResearch Laboratories, West Grove, PA) or Alexa488 (1:200, Jackson ImmunoResearch Laboratories, West Grove, PA) conjugated antibodies and counterstained with 4´,6-diamidino-2-phenylindole (DAPI; 1:5000, Invitrogen, Burlington, ON). Numbers of CXCR7-positive cells were determined in at least 10 standardized microscopic fields of 10.000 μm2 each defined by an ocular morphometric grid as indicated in the text and figures as the mean number of cells/μm^2^ ± SEM. For statistical analysis, a Bonferroni-corrected one-way ANOVA tests was performed. The test was classified as significant if the p-value was <0.05 (GraphPad PRISM). All images were taken on an Olympus fluorescent microscope. None of the study authors was involved in decision-making with respect to biopsy. The study was approved by the Ethics Committee of the University of Münster.

**Table 1:** Human brain tissues used for immunohistochemical analysis.

	Diagnosis of biopsy	Sampling location	Age/Sex
1	Brain tissue with reactive changes	right frontal	72/m
2	Brain tissue with reactive changes	right paraventricular	66/f
3	Brain tissue with reactive changes	right parieto-occipital	25/f
4	Brain tissue with reactive changes	unknown	51/m
5	Brain tissue with reactive changes	right parietal	72/m
6	Brain tissue with reactive changes	unknown	47/f
7	Inflammatory demyelinating lesion consitent with MS	right frontal	44/f
8	Inflammatory demyelinating lesion consitent with MS	left frontal	74/f
9	Inflammatory demyelinating lesion consitent with MS	supraventricular	50/f
10	Inflammatory demyelinating lesion consitent with MS	subcortical	41/f
11	Inflammatory demyelinating lesion consitent with MS	right occipital	26/f
12	Inflammatory demyelinating lesion consitent with MS	left temporal	21/f
13	Inflammatory demyelinating lesion consitent with MS	left parieto-occipital	63/f
14	Inflammatory demyelinating lesion consitent with MS	cerebellar	25/f
15	Inflammatory demyelinating lesion consitent with MS	leukocortical	34/f
16	Inflammatory demyelinating lesion consitent with MS	cerebellar	48/m
17	Inflammatory demyelinating lesion consitent with MS	semioval center	45/m
18	Inflammatory demyelinating lesion consitent with MS	left parieto-occipital	61/f

## Results

### CXCR7 is expressed in mature NogoA positive oligodendrocytes and Nkx2.2 positive OPCs in remyelinating MS lesions

**Fig 1:**
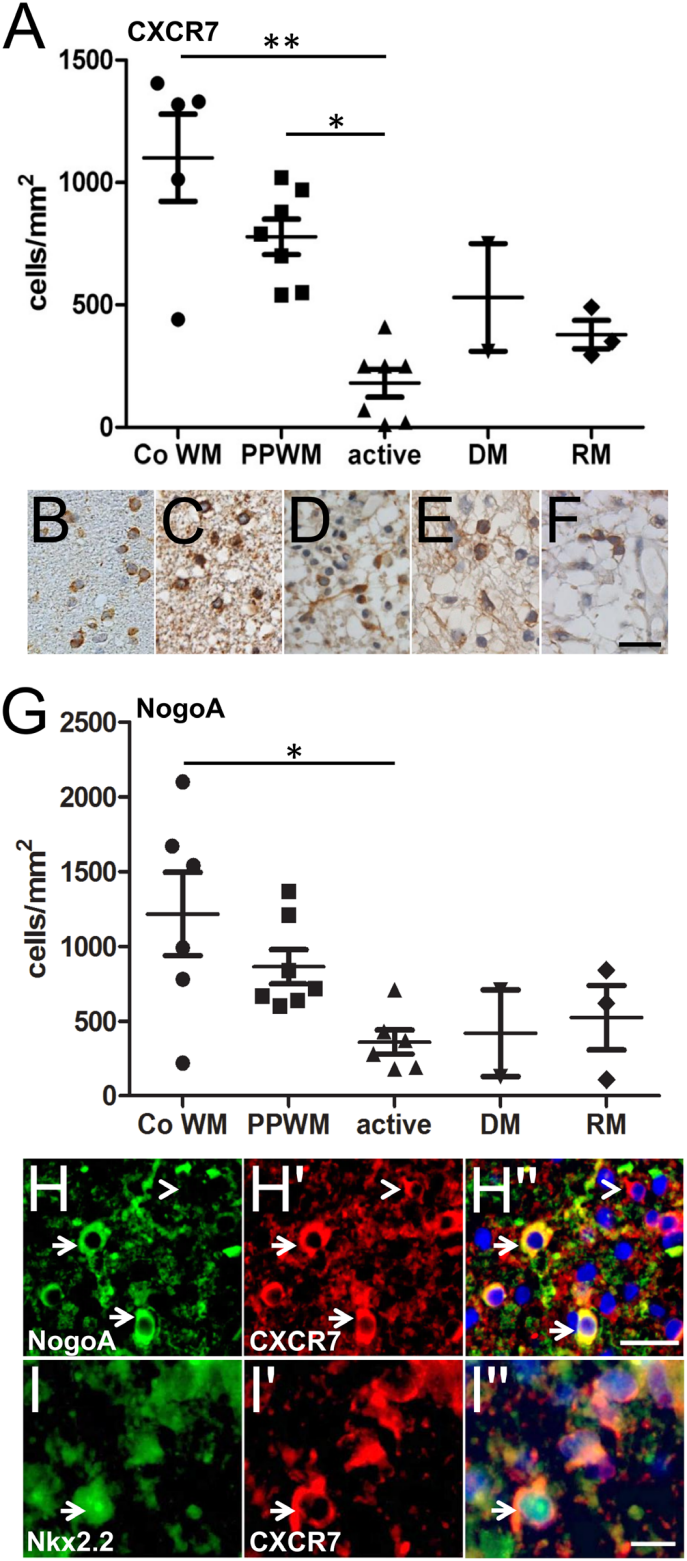
CXCR7 detection in MS tissue. (A-F) CXCR7 staining revealed receptor expression on cells in control brains (B) and different regions of the MS brain including demyelinated (E; DM) and remyelinating (F; RM) lesions as well as active white matter lesions (D). Most CXCR7-positive cells in the MS brain were detected in the periplaque white matter (C; PPWM) while their number was significantly decreased in actively demyelinating lesion areas (D; active) at the plaque border probably reflecting ongoing oligodendroglial cell death (one-way ANOVA, **p < 0,05). (G) NogoA staining of control and MS brains demonstrated a similar distribution as CXCR7 (A) with a correlation coefficient of 0,783 between stainings. Double immunostaining then confirmed that mature NogoA positive oligodendrocytes (H-H''; see arrows) and Nkx2.2 positive oligodendroglial precursor cells (I-I''; arrows) express CXCR7. Not all CXCR7-positive cells were of oligodendroglial origin which is in line with studies describing this receptor in other CNS cell types (see arrowhead in H-H''). Scale bars: 25μm (F), 25μm (H-H''), 10μm (I-I'').

Immunohistochemistry of human MS tissue revealed that CXCR7-expressing cells are present in control brains and different areas of the MS brain (Fig 1A–1F). While in the periplaque white matter (PPWM) an average of 750 CXCR7-expressing cells per mm^2^ could be detected this number was significantly decreased to approximately 200 cells/mm^2^ in actively demyelinating lesion areas (active) at the plaque border. As these areas were infiltrated by numerous macrophages containing myelin degradation products this drop in numbers probably reflects ongoing oligodendroglial cells death—a common observation in MS pathology. The number of CXCR7-expressing cells was also lower in demyelinated (DM) areas featuring macrophages and T cells not containing myelin degradation products (approx. 500 cells/mm^2^) and remyelinating (RM) areas characterized by newly formed thin and irregular myelin sheaths (approx. 400 cells/mm^2^). In order to clarify the identity of the CXCR7-expressing cells in the above-described regions of the MS brain we then performed stainings of NogoA, a reliable marker for mature oligodendrocytes [20], of the same regions in parallel sections (Fig 1G). This revealed that the distribution of NogoA largely mirrored that of CXCR7 with an overall correlation coefficient of 0,783. Double immunofluorescent labeling of CXCR7 in combination with Nogo-A (see arrows in Fig 1H–1H'') and Nkx2.2, a transcription factor strongly expressed in the nucleus of OPCs (see arrow in Fig 1I–1I''; [5]) then confirmed that both mature and precursor cells in remyelinating MS lesions express CXCR7. Of note—and as expected since expression of CXCR7 has been described in several CNS cell types such as astrocytes [21, 22]—not all of the detected CXCR7-expressing cells belonged to the oligodendroglial lineage (see arrowhead in Fig 1H–1H'').

### CXCL12 stimulation promotes the expression of oligodendroglial differentiation markers

**Fig 2:**
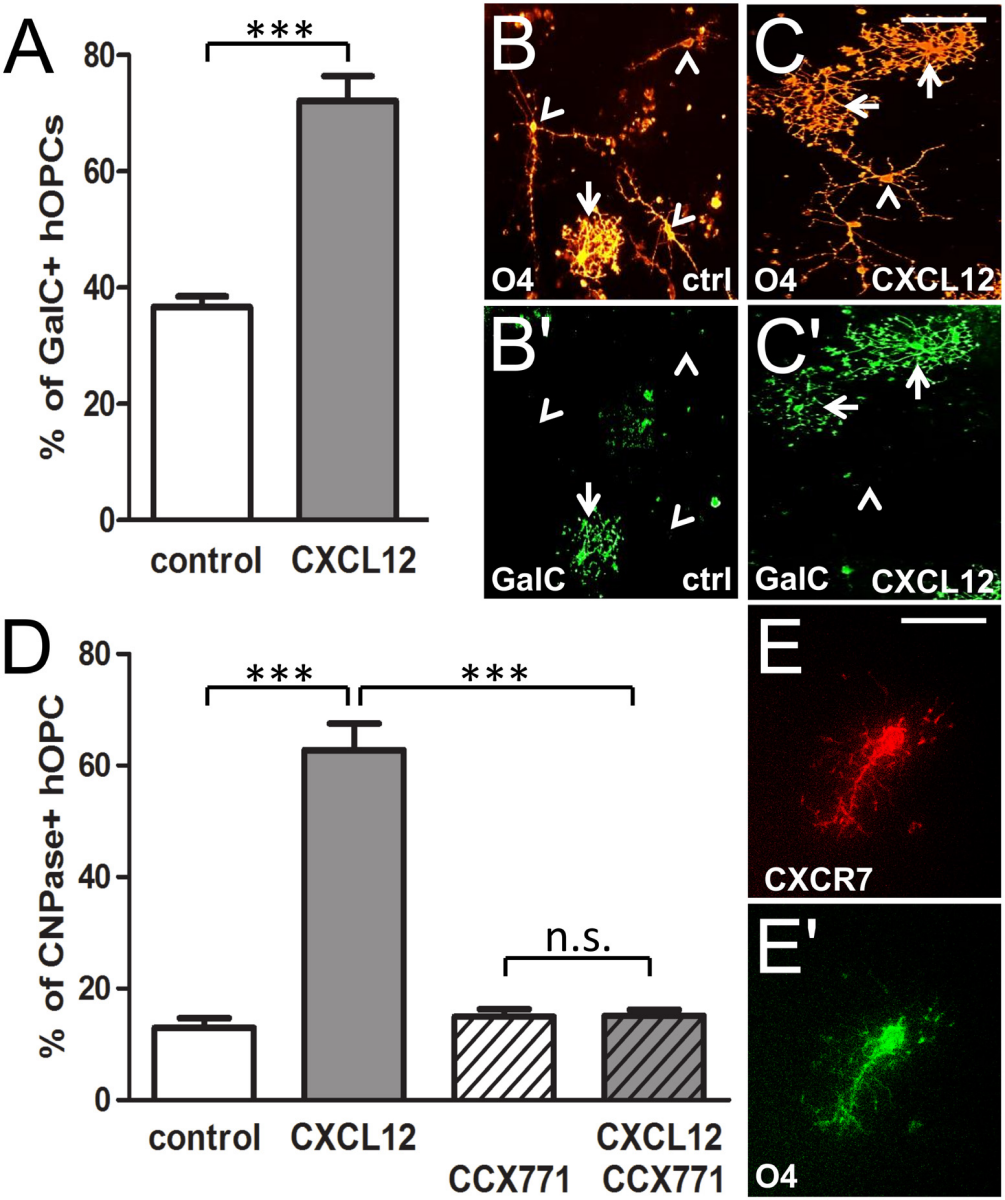
CXCL12-mediated promotion of differentiation marker expression in cultured human oligodendroglial precursor cells. (A) The percentage of O4 positive cells expressing GalC was significantly increased after 7 days of CXCL12 stimulation as compared to control cells. (B-C') Representative immunostainings of GalC/O4 positive OPCs stimulated with CXCL12 and control cells. Note that in CXCL12-stimulated cultures complex cell morphologies (arrows) were more common than in control cultures (arrowheads indicating cells with fewer cellular processes). (D) Determination of the percentage of CNPase positive hOPCs under CXCL12 stimulation after 7 days. This demonstrated that the CXCL12-dependent promotion of myelin induction (white vs. gray bars) was completely abolished in the presence of CCX771 (dashed gray bar). CCX771 alone did not affect myelin expression (compare white to dashed white bar). (E-E') Double immunostaining revealed that O4 positive precursor cell express CXCR7. Data are shown as mean values +/- SEM derived from 3 independent experiments. (t-test, ***p <0,001 and ANOVA, **p < 0,01). Scale bars: 70 μm (B-C'), 30 μm (E-E') μm.

For a functional analysis of CXCL12-dependent effects on human OPCs, we exposed A2B5 antibody selected fetal human precursor cells to this chemokine. As oligodendroglial maturation is reflected by the induction of specific lineage markers, we determined whether expression kinetics was altered upon CXCL12 stimulation (Fig 2). To this end, fetal progenitors from the Human Fetal Tissue Repository were stimulated with 100ng/ml recombinant CXCL12 for 7 days, fixed, and subjected to immunofluorescent staining with antibodies directed against O4 and GalC. The assessment of OPC differentiation based on double positivity for O4 and GalC is widely used in the literature as a maturation read-out [23, 24] and revealed here that after 7 days of CXCL12 stimulation, the percentage of O4 cells that expressed GalC was increased more than 2-fold, compared to controls (Fig 2A–2C'). In parallel to our previous study investigating the effects of CXCL12 on rodent OPCs [15] most CXCL12-stimulated hOPCs featured more complex morphologies (arrows) as compared to cells in control cultures (arrowheads). Of note, with regard to potential effects of CXCL12 on hOPC proliferation and survival, DAPI counts of cells at different time points revealed no significant difference in cell numbers between stimulated or non-stimulated cells (data not shown). To determine whether the observed CXCL12-dependent differentiation effects were mediated through CXCR7 and in order to reproduce and corroborate the previous results in hOPCs from a different source, we then conducted specific blocking experiments with commercially available hOPCs. Using the expression of CNPase as a readout, we applied the specific CXCR7 antagonist CCX771. When applied alone (dashed white bar), CCX771 did not affect the degree of CNPase positive hOPCs (Fig 2D), whereas CXCL12 application (grey bar) boosted CNPase expression 3-fold similar to our previous observations using GalC-positivity of hOPCs from the Human Fetal Tissue Repository. Of note, this induction was completely abolished in CXCL12-stimulated OPCs that had been pre-treated with CCX771 (dashed grey bar), suggesting a crucial role for CXCR7 in the transmission of oligodendrocyte differentiation signals. In order to rule out potential DMSO-associated effects we also stimulated hOPCs with DMSO alone (white bar). In addition, CXCL12 stimulation was also carried out in the presence of DMSO to provide an appropriate control for CCX771 inhibition. Furthermore, double immunostaining confirmed that O4 positive hOPCs expressed CXCR7 (Fig 2E and 2E’).

### CXCL12 stimulation leads to enhanced morphological maturation of CXCR7-positive human OPCs

**Fig 3:**
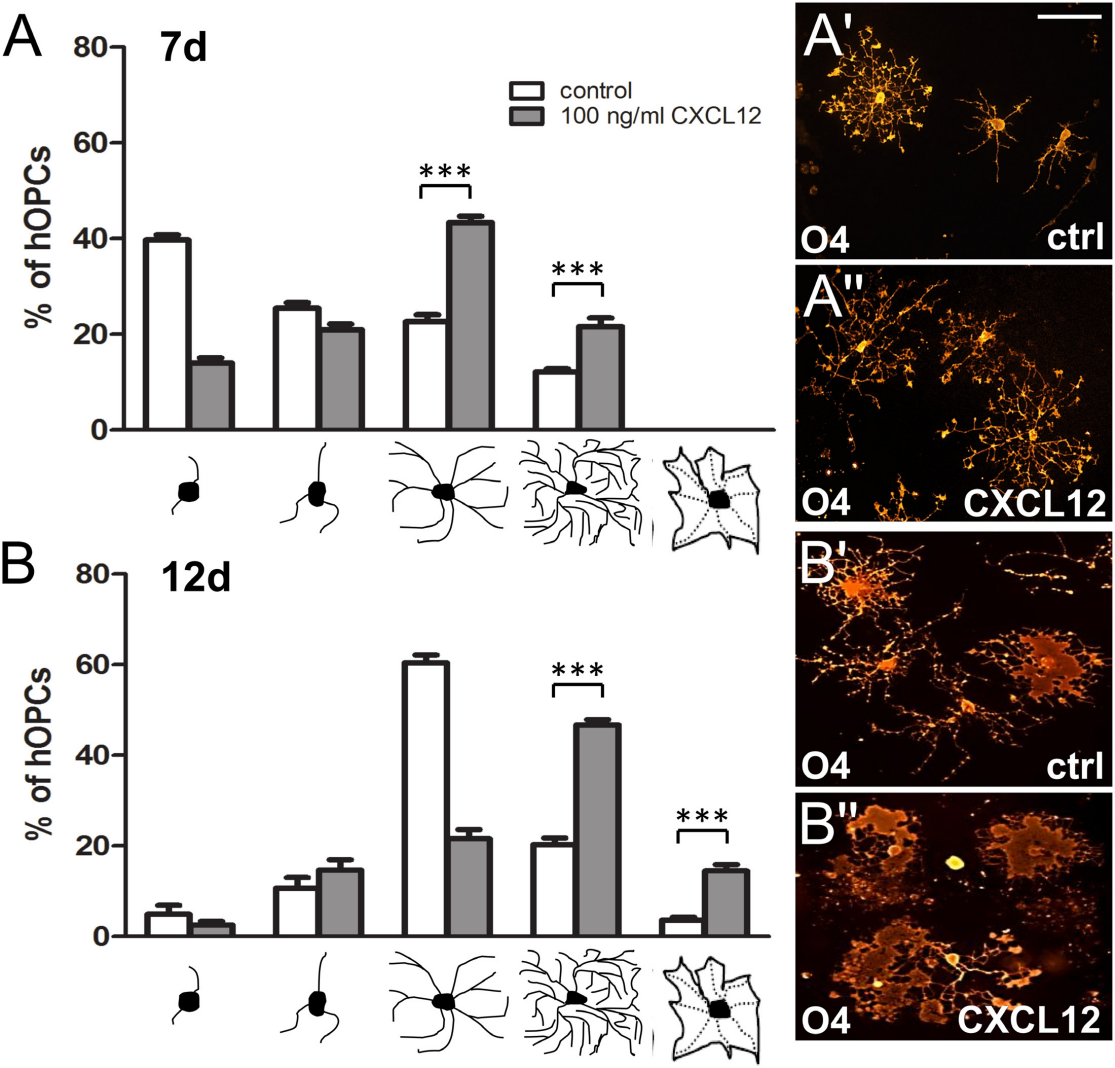
CXCL12 promotes morphological maturation of cultured human oligodendroglial precursor cells. Five different morphologies were distinguished ranging from a very low number of processes in precursor cells to multiple process-bearing cells (low, medium, high) to mature cells with a very high degree of arborization or flattened appearance (membrane sheet). (A,B) Analysis of hOPC morphology distribution revealed a CXCL12-dependent shift towards more mature cells (white bars: buffer treated cells; gray bars: CXCL12-stimulated cells) after 7 and 12 days in culture. Data are shown as mean values +/- SEM derived from 3 independent experiments (ANOVA, **p < 0,01). Representative O4-expressing hOPCs stimulated with either buffer or CXCL12 at 7d (A',A'') and 12d (B',B''). Scale bars: 50μm.

As oligodendrocyte lineage marker staining of control versus CXCL12-stimulated hOPCs suggested that cell shape and size are also influenced by this chemokine, we determined quantitatively whether hOPC morphological maturation is promoted as well (Fig 3). For visualization of single cells, we stained hOPCs from the Human Fetal Tissue Repository with the previously used anti-O4 antibody. Differentiation of cultured hOPCs is not a synchronized process and heterogeneous cell populations are generally observed featuring various degrees of morphological maturation with increasing numbers of processes and branches. In order to classify cell morphology we therefore used a well-established grading system that was already introduced in several previous studies [25–27] which distinguishes between five different morphologies ranging from a very low number of processes in precursor cells to multiple process-bearing cells and mature cells with a very high degree of arborization or membrane sheet appearance (see below graphs in Fig 3). We initiated oligodendroglial differentiation and applied recombinant CXCL12 or buffer as control condition, and fixed cells after 7 (Fig 3A–3A'') and 12 days (Fig 3B–3B''), respectively. This clearly demonstrated that application of CXCL12 resulted in a significant shift towards more complex hOPC morphologies as compared to control (buffer stimulated) cells. At 7 days, the majority of control cells featured low and medium morphologies, whereas the majority of cells stimulated with 100ng/ml CXCL12 were already at a highly differentiated stage. At 12 days CXCL12 stimulation resulted in an additional shift, with the majority of cells now displaying membrane sheet structures (see Fig 3B'').

## Discussion

Inefficient CNS remyelination observed in neuroinflammatory demyelinating diseases such as MS is tightly linked to the limited ability of resident OPCs to differentiate properly—a prerequisite for subsequent remyelination [5, 28]. While previous studies have demonstrated CXCR7 expression in human adult neurons [29], brain tumors [30] and human brain microvascular endothelial cells (HBMECs, [31]), we here demonstrate that CXCR7 is expressed on oligodendroglial cells in MS brain lesions and that its activation by CXCL12 leads to an acceleration of human glial maturation in culture as demonstrated by expression of oligodendrocyte lineage markers and enhanced morphological maturation. Regarding the relevance of CXCL12 *in vivo* several groups have already demonstrated that in the MS brain CXCL12 is expressed by reactive astrocytes particularly near the lesion edge [32] and that the proinflammatory cytokine interleukin-1β and myelin debris in the form of MBP can induce astrocytic CXCL12 [33]. With respect to the underlying mechanisms of CXCR7 signaling our previous studies have demonstrated that CXCR7 activation on oligodendroglial cells by CXCL12 leads to ERK1/2 phosphorylation [15] which is in line with several other studies indicating that factors involved in oligodendroglial differentiation and myelination exert their effects through the ERK1/2 signaling cascade [34–36]. Nevertheless, the mechanisms that promote oligodendrocyte differentiation upon ERK phosphorylation are still largely elusive even though a recent study revealed that both ERK1 and -2 promote MBP gene expression in oligodendroglial cells via phosphorylation of the transcription factor Sp1 [37]. On the other hand, other groups have clearly shown that G protein-coupled receptor kinase 2 (Grk2) is essential for CXCR7 signaling in CNS cells [21]. In how far Grks also play a role in oligodendroglial CXCR7 signaling is currently unclear and needs to be further elucidated in future studies on human tissue.

Of note, related studies have linked CXCL12-mediated OPC maturation to signaling through its second well-described receptor CXCR4. However, the experiments in these studies were performed in the non-inflammatory toxic cuprizone animal model [38] which makes a potential translation to MS with its strong inflammatory component difficult. In contrast the data presented here were generated in the human paradigm and demonstrate for the first time that CXCR7 is present on OPCs and oligodendrocytes in the MS brain. In addition, Williams and colleagues used the controversial substance AMD3100, a compound initially developed as a specific CXCR4 inhibitor. Pharmacological studies have, however, demonstrated that this molecule cannot be used as a reliable and specific blocking reagent as it was shown to act as a weak partial CXCR4 agonist leading, for instance, to an intracellular calcium levels increase similar to CXCL12 [39].

Nonetheless, CXCR4 might still play a role in CXCR7-mediated OPC maturation as several studies demonstrated receptor heterodimerization [40, 41] being relevant for CXCL12-mediated signal transmission. Of note, the goal of this study was not to demonstrate an exclusive responsibility of CXCR7 for OPC maturation but rather aimed at proving that CXCR7 is critically involved in this process thus providing evidence that this receptor could by a target for therapeutic repair. Yet, the exact downstream signaling of such heterodimers is still controversially discussed since some studies indicated, for instance, that coexpression enhances Ca^2+^ mobilization or chemotaxis [41, 42] while others demonstrated compromised CXCR4 signaling [40]. We and others support the idea that CXCR7 signals independently from CXCR4 and the results of our specific blocking experiments presented here have clearly demonstrated that CXCR7 is highly relevant for human OPC differentiation. This is of even greater interest since recently two small-molecules, VUF11207 and VUF11403, were identified as highly potent and selective ligands for CXCR7 that induce recruitment of β-arrestin [43]. These new compounds might represent promising lead substances for the development of specific CXCR7-based remyelination strategies with the benefit of avoiding CXCR4-mediated effects. However, a prerequisite for such therapies is a sufficient presence of CXCR7 on target cells and as we found that CXCR7 positivity undergoes an overall decrease in MS tissue in comparison to controls this is a critical aspect with potential therapeutic implications. In general, it is conceivable that the overall decrease of CXCR7-positive cells may partly reflect oligodendroglial cell death. However, CXCR7 positivity increases again in remyelinating lesions possibly based on migration of recruited OPCs. During this phase of lesion repair a window of opportunity might open up allowing for specific CXCR7-based OPC stimulation. However, lesion repair is overall insufficient which is thought to be partly due to the expression of chemorepellent molecules in MS lesions such as, for instance, Semaphorin 3A. Thereby, instead of entering MS lesions OPCs could be arrested in the periplaque white matter where, accordingly, we found significantly more CXCR7-positive cells in comparison to lesions themselves. This suggests that while CXCR7 activation could be an overall beneficial cue for remyelination in MS the key prerequisite for such an effect is successful entry of OPCs into MS lesions. We therefore conclude that besides and beyond a potential exogenous CXCR7 receptor upregulation to facilitate repair future combinatorial therapies are required that stimulate both OPC migration and differentiation to ultimately induce lesion repair.
